# Intra-Abdominal Pressure Correlates with Extracellular Water Content

**DOI:** 10.1371/journal.pone.0122193

**Published:** 2015-04-07

**Authors:** Wojciech Dąbrowski, Edyta Kotlinska-Hasiec, Andrzej Jaroszynski, Przemyslaw Zadora, Jacek Pilat, Ziemowit Rzecki, Wojciech Zaluska, Daniel Schneditz

**Affiliations:** 1 Department of Anesthesiology and Intensive Therapy Medical University of Lublin, Lublin, Poland; 2 Department of Family Medicine Medical University of Lublin, Lublin, Poland; 3 Department of General Surgery, Transplantology and Clinical Nutrition Medical University of Lublin, Lublin, Poland; 4 Department of Nephrology Medical University of Lublin, Lublin, Poland; 5 Department of Physiology, Medical University of Graz, Graz, Austria; Bambino Gesù Children's Hospital, ITALY

## Abstract

**Background:**

Secondary increase in intra-abdominal pressure (IAP) may result from extra-abdominal pathology, such as massive fluid resuscitation, capillary leak or sepsis. All these conditions increase the extravascular water content. The aim of this study was to analyze the relationship between IAP and body water volume.

**Material and Methods:**

Adult patients treated for sepsis or septic shock with acute kidney injury (AKI) and patients undergoing elective pharyngolaryngeal or orthopedic surgery were enrolled. IAP was measured in the urinary bladder. Total body water (TBW), extracellular water content (ECW) and volume excess (VE) were measured by whole body bioimpedance. Among critically ill patients, all parameters were analyzed over three consecutive days, and parameters were evaluated perioperatively in surgical patients.

**Results:**

One hundred twenty patients were studied. Taken together, the correlations between IAP and VE, TBW, and ECW were measured at 408 time points. In all participants, IAP strongly correlated with ECW and VE. In critically ill patients, IAP correlated with ECW and VE. In surgical patients, IAP correlated with ECW and TBW. IAP strongly correlated with ECW and VE in the mixed population. IAP also correlated with VE in critically ill patients. ROC curve analysis showed that ECW and VE might be discriminative parameters of risk for increased IAP.

**Conclusion:**

IAP strongly correlates with ECW.

## Introduction

Intra-abdominal hypertension (IAH) defined as intra-abdominal pressure (IAP) above 12 mmHg, is a serious problem in critically ill patients. Primary IAH originates from events in the abdominal region such as massive intra-abdominal bleeding, surgical “packing”, ileus, or acute pancreatitis, whereas secondary IAH has an extra-abdominal origin such as in severe burn patients, after massive fluid therapy, or in septic shock patients [1–4]. Indeed, increased microvascular permeability results in massive fluid shifts into the interstitial space, thus increasing the extravascular water content and subsequently resulting in intestinal edema [5].

Fluid removal is an important treatment in patients with renal insufficiency and in critically ill patients with acute kidney injury (AKI). Conventional intermittent daily hemodialysis is typically used to treat AKI, but continuous veno-venous hemofiltration (CVVH) is becoming increasingly popular [6]. The major advantages of CVVH are slow and continuous fluid removal, cardiovascular stability, and improved metabolic control [7,8]. Unfortunately, the volume of removed fluid is mainly determined in accordance with hemodynamic status. Yet, a sensitive marker of fluid removal has been not identified.

Accurate assessment of fluid status in patients with AKI is difficult and frequently based on clinical symptoms. Several methods have been reported to determine volume excess (VE) in patients with AKI with limited sensitivity and clinical feasibility [9,10]. Whole body bioimpedance analysis (BIA) is a safe, noninvasive, and practical tool to measure body fluid compartments, and it is easily applied to everyday practice. Several studies have presented its usefulness to monitor body water changes during renal replacement therapy [11–13]. Whole body BIA may provide useful information not only in well-established patients who require hemodialysis but also in critically ill patients [11,14,15]. However, the interpretation of the data is more difficult. The raw impedance data measuring resistance and reactance at specified frequencies provide only indirect information about the total and extracellular water content (TBW and ECW, respectively). In practical applications, however, the physician faces the problem of estimating volume deficits and VE.

The aim of the present study was to determine whether IAP was related to TBW, ECW, and VE in a mixed population of patients.

## Methods

### Ethics Statement

This prospective observational study was conducted in adult patients undergoing elective pharyngolaryngeal or orthopedic surgery and in critically ill patients. The study was conducted in accordance with the Declaration of Helsinki and was approved by the Institutional Review Board and the Bioethical Committee for Human Studies of Medical University at Lublin, Poland (KE-0254/58/2010). Written informed consent was obtained from all participants or from the legal representatives of patients when patients were sedated and mechanically ventilated.

### Surgical patients

IAP, VE, TBW, and ECW were measured in patients undergoing pharyngo-laryngeal surgery under general anesthesia or orthopedic surgery under spinal anesthesia at the following four time points: just before surgery, just after surgery, 3 hours after surgery and on the morning of the first postoperative day. Patients received infusions of crystalloids during surgery and in the early postoperative period. The dose of fluids depended on the patient’s hemodynamic status.

### Intensive care patients

Critically ill adult patients treated for acute respiratory insufficiency, sepsis, or septic shock complicated by AKI were enrolled. Sepsis was defined using the criteria described by Bone et al. [16]. The diagnosis of AKI was based on RIFLE criteria [17]. CVVH was used in patients with anuria or severe oliguria who did not respond to furosemide. In patients who responded to furosemide, a continuous infusion (120 to 240 mg per day) was used. IAP, VE, TBW, and ECW were measured at the day of admission into the intensive care unit (ICU) and at 24 and 48 h after the admission into ICU.

### Data collection and study protocol

IAP was measured twice daily via a urinary bladder Foley catheter, with a maximal priming solution of 25 ml of sterile saline, in the complete supine position with the transducer zeroed at the level of the mid-axillary line.

VE, TBW, and ECW were measured by whole-body BIA using the Body Composition Monitor (BCM, Fresenius Medical Care, Hamburg, Germany). Prior to examination, the height and body mass of the patient were measured. Electrodes were placed on the wrist (proximally to the metacarpophalangeal joint) and ankle (proximally to the transverse metatarsal arch on the superior side of the foot). Bioimpedance was measured at 50 frequencies ranging from 5 kHz to 1 MHz in a supine body position.

In patients undergoing orthopaedic surgery electrodes were placed on the body side opposed to the surgery site to avoid effects of local tissue edema.

### Statistics

The Spearman rank correlation test was used for the overall analysis. A p-value < 0.05 was considered significant to reject the null-hypothesis. Cut-off points were calculated with the use of receiver-operator characteristics (ROC) with auto-calculated maximum specificity and sensitivity.

## Results

Forty-eight patients underwent pharyngolaryngeal or orthopedic surgery, and 72 patients were treated for severe sepsis or septic shock with AKI (Table 1). Taken together, the correlations between IAP and VE, TBW and ECW were measured at 408 time points (192 time points among surgery patients and 216 time points among critically ill patients).

**Table 1:** Demographic data.

Patients		Female	Male	Age (years)
Studied population		38	82	57 ± 16
(n = 120)				
Critically ill patients		29	43	56 ± 15
(n = 72)				
Surgical patients (n = 48)	Orthopaedic patients (n = 25)	7	18	55 ± 21
	Pharyngolaryngeal patients (n = 23)	2	21	62 ± 7

**Fig 1:**
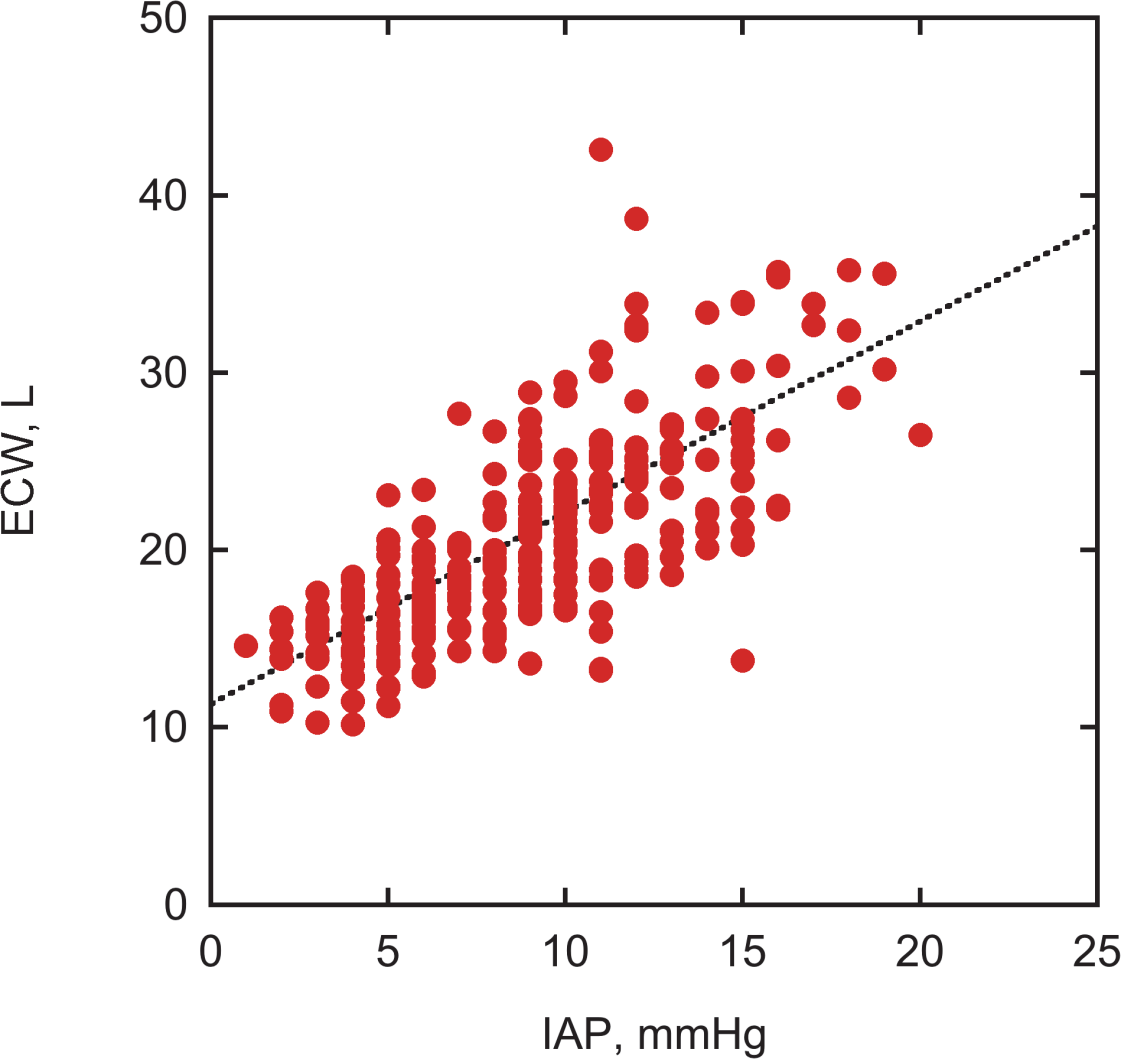
Correlation between intra-abdominal pressure (IAP) and extracellular water content (ECW) in all participants (Spearman correlation—p < 0.001, r = 0.74).

**Fig 2:**
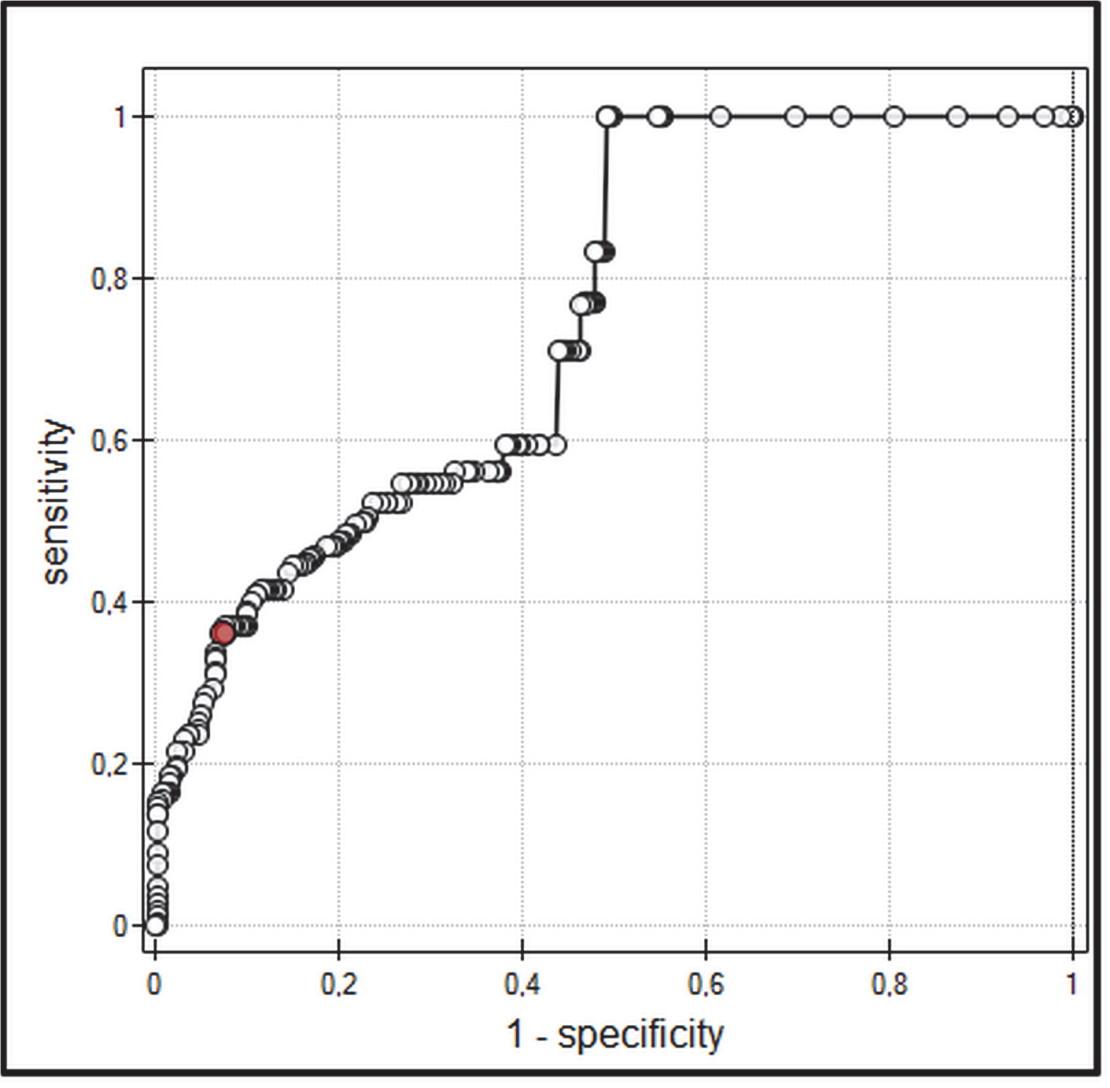
ROC curve for extracellular water content (ECW) as a diagnostic test of intra-abdominal hypertension (IAH). Cut-off point of ECW for IAH = 23.9 L. Area under the curve (AUC) = 0.87.

**Fig 3:**
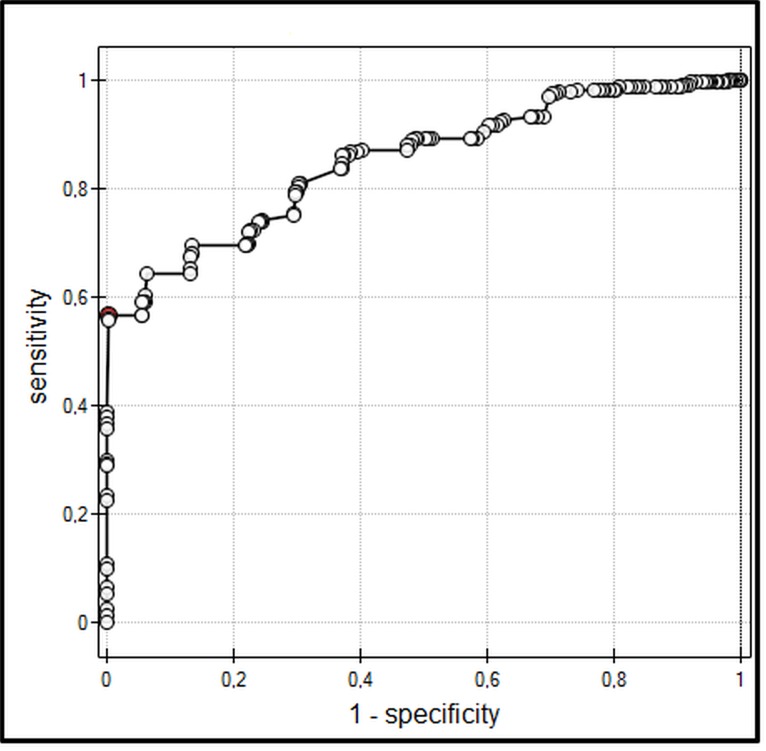
ROC curve for volume excess (VE) as a diagnostic test of intra-abdominal hypertension (IAH). Cut-off point of VE for IAH = 6.6 L. Area under the curve (AUC) = 0.85.

In all participants, IAP strongly correlated with ECW (Fig. 1). Moreover, IAP correlated with VE and TBW (p < 0.001, r = 0.67). ROC analysis showed that ECW and VE might be discriminative parameters in risk of IAH. 23.9 L of ECW and 6.6 L of VE were calculated as a cut-off points for the development of IAH in the studied population (Figs. 2 and 3). IAH developed in 28% of all patients (47% in ICU, and 1% in surgery patients, respectively).

In critically ill patients, IAP ranged between 4 and 20 mmHg. There were 101 episodes of IAH. ECW ranged between 12.2 and 35.8 L, TBW ranged between 24.1 and 82.8 L and VE between 0.2 and 15.7 L. In critically ill patients IAP correlated with ECW, VE, and TBW, and 22.4 L of ECW and 6.6 L of VE were calculated as the cut-off points for the development of IAH (Table 2).

**Table 2:** Correlation between intra-abdominal pressure (IAP) and total body water content (TBW), extracellular water content (ECW) and volume excess (VE) and cut-off point for all these parameters in prediction of IAH (IAP higher than 12 mmHg).

Patients	Parameter		Spearman correlation	Cut-off point
				(AUC)
Critically ill patients	IAP (mmHg)	ECW	p < 0.001, r = 0.62	22.4 (L)
				(0.800)
		TBW	p < 0.001, r = 0.55	52.2 (L)
				(0.761)
		VE	p < 0.001, r = 0.64	6.6 (L)
				(0.801)
Surgical patients		ECW	p < 0.001, r = 0.65	24.9 (L)
				(0.848)
		TBW	p < 0.001, r = 0.47	53.6 (L)
				(0.695)
		VE	p < 0.001, r = 0.35	9.5(L)
				(0.713)

AUC—area under the receiver-operator characteristic.

In surgical patients, IAP ranged between 1 and 15 mmHg. Ten episodes of IAH were noted 3 h after surgery. ECW ranged between 10.2 and 42.7 L, TBW ranged between 25.6 and 63.9 L, and VE ranged between -4.4 and 10.5 L. There was a strong correlation between IAP and ECW, and 24.9 L of ECW and 9.5 L of VE were calculated as a cut-off points for development of IAH (Table 2).

## Discussion

This study shows that IAP significantly increased as ECW increased in critically ill patients and in patients undergoing pharyngolaryngeal or orthopedic surgery. Moreover, IAP correlated with TBW. In critically ill patients, IAP also correlated with VE. The risk of IAH significantly increased when ECW volume exceeded 22.4 L in critically ill and 24.9 L in surgical patients.

IAH has been observed to occur in 18 to 83% of septic shock patients and in 12 to 40% of patients following major surgery [18–20], and it may result from a positive fluid balance associated with elevated vascular permeability. An increase in capillary leakage following a stress-related inflammatory response leads to extravascular fluid accumulation, which is likely to result in gastrointestinal tract edema and increased IAP [21,22]. Edema is caused by several physiological changes such as: 1) increased capillary pressure leading to stasis edema, 2) blood dilution and a decrease in colloid osmotic pressure leading to hypoproteinemic edema, 3) increased microvascular permeability leading to permeability edema and 4) decreased lymph flow leading to cold lymph edema [21]. Positive cumulative fluid balance increases extravascular water, leading to IAH [23]. Our present and previous studies unambiguously confirmed these ideas [11]. The strong correlation between IAP and ECW demonstrates that the level of IAP strongly depends on ECW.

The measurement of fluid volume and tissue hydration by bioimpedance has great potential in critically ill patients and in anesthesiology to measure excessive administration of fluid that is used to maintain systemic blood pressures. Bioimpedance analysis draws upon the bioelectrical properties between the wrist and ankle under the assumption of a steady fluid distribution between different body compartments. Unfortunately, the interpretation of data is more difficult. In the current analysis, the body can be thought to consist of three segments (the arm, trunk, and leg) with different cross-sectional areas and lengths. The geometrical differences of these three segments importantly influence their contribution to overall whole-body bioimpedance [24]. Importantly, the same amount of extracellular fluid can lead to different changes in whole-body bioimpedance, depending upon whether this fluid is added to or removed from the leg, arm, or trunk. Extracellular volume accumulating in the trunk such as with non-essential ascites, hemothorax, or inflammatory-related lung interstitial edema is only incompletely measured by whole-body BIA. Whole-body BIA is much more sensitive to volume changes in the limbs [25]. An increase in IAP above 20 mmHg not only causes venous congestion in visceral organs but also reduces the venous outflow from the lower limbs, thus increasing femoral venous pressure [26]. This pathology may increase extravascular water in the limbs thereby overestimating overall fluid volumes. In our study, we did not observe IAP greater than 20 mmHg. Only three of the critically ill patients had IAP equal to 20 mmHg, and their exclusion from the statistical analysis did not significantly change the correlation between IAP and ECW and VE (data not shown). Therefore, we conclude that IAP is strongly correlated with ECW, as measured by whole body bioimpedance among patients with IAH lower than 20 mmHg.

Despite the promise of novel findings, a few limitations should be discussed. First, the number of patients with IAP higher than 15 mmHg (II grade of IAH in accordance with World Society of Abdominal Compartment Syndrome, www.wsacs.org) was small and all of these patients were treated for septic shock. Increased IAP above 15 mmHg is suspected to affect the respiratory system leading to hypoxia and hypercapnia [3,27]. This pathology requires to increase inspiratory and/or end-expiratory pressures during artificial ventilation. Both pressures, however, independently affect IAP, reduce venous return, and induce venous congestion [27,28]. Secondly, ECW, TBW, and VE were measured in critically ill patients treated with CVVH due to acute kidney injury. Renal replacement therapy initially removes fluids from central compartments thereby blunting the whole body BIA changes [29]. We therefore assume that the correlation between IAP and body water distribution is less reliable in the acute setting during the first and second days of CVVH. Last, but not last, the volumes and thresholds determined in this study apply to the average adult patient. Normalizing volumes to body size is likely to provide even better ROC parameters but this remains to be analyzed in a larger cohort of patients with a larger spread in body sizes.

Finally, we demonstrated that IAP strongly correlated with ECW in critically ill patients and patients undergoing extra-abdominal surgery. Based on our findings, we propose IAP to be a marker of ECW.
